# Observations on Dysmenorrhœa

**Published:** 1847-09

**Authors:** 

**Affiliations:** Lecturer on Midwifery at Guy’s Hospital


### SELECTIONS

FROM

### AMERICAN AND FOREIGN JOURNALS.

---

*Observations on Dysmenorrhœa.* By Dr. Oldham, Lecturer on Midwifery at Guy's Hospital.—Dr. Oldham considers that there are two forms of dysmenorrhœa, which admit of being well defined; one in which the os uteri is too narrow, and the flow of the menses consequently impeded; and another form which is distinguished by a membrane being cast off from the cavity of the womb. The former he calls mechanical dysmenorrhœa, the latter membranous dysmenorrhœa. He illustrates the first form by two cases which may be thus briefly mentioned.

Case I.—A married woman, æt. 31 years, married two years, but never pregnant, consulted Dr. Oldham for painful and scanty menstruation, and more particularly for sterility, on account of which she had been reproached by her husband. On examination the uterus was found natural, with the exception of the os, which was extremely small and contracted. Dr. Oldham resolved in this case to dilate the os by means of Dr. Simpson's concealed bistoury, which was accomplished without pain and without bleeding. A small-sized sponge tent, coated with tallow, was then fixed in the part, and on the following day a common metallic bougie was passed and repeated at intervals of a week, the size being gradually increased. After this treatment, she passed through a period with much less pain than usual, and eventually became pregnant.

Case II—was a woman, ætat. 32 years, complaining of scanty and painful menstruation. The pains were described as peculiarly severe during the period. After fruitless attempts to cure her by sedatives and attention to the general health, Dr. Oldham divided the os, as in the former case, but the relief was not so decided.

Upon these cases the author remarks as follows:

It will be seen that in the first case the benefit of an artificial section was most marked. Immediate relief to the pain of menstruation, an increased flow, and a cure of the sterility followed upon it. In the second case, the dilatation afforded only partial relief. The first case proves that the operation recommended by Dr. Macintosh is legitimate and useful; and that by it we may, so to speak, release the functions of the womb from a restraint which is essentially morbid. Taking the two cases together, there is also another inference, that the simple presence of a small os uteri is not enough to warrant its performance. I believe that the main distinction in the two classes of cases is, that in the one there is a full development of the internal organs, with the exception of the morbidly small outlet of the womb; in the other, the whole sexual system is but imperfectly developed. This is ascertained by measurement by Simpson's uterine sound, and by the weight of the organ as ascertained by the finger.

There is a symptom common to the two cases which demands a word, viz: the scantiness of the flow. I believe Dr. Churchill to be right when he says that in dysmenorrhœa the discharge may be scanty, profuse, or in ordinary quantity; but it appears to me, that this fact implies another, namely, that there is a corresponding variation in the pathological states which accompany them. In the two cases related, there is to be found the explanation of the diminished flow, in the physical imperfection of the uterus itself. Cases of the second form ought to be classed with cases of amenorrhœa.

The author concludes this portion of his communication with the following summary of his views:

1. There is a form of difficult menstruation which depends upon a morbid contraction of the sexual passages, which may be either congenital or acquired, and affect either the os uteri internum or externum.

2. That this form of dysmenorrhœa is usually accompanied with a scanty flow of the menses and sterility.

3. That a great practical distinction is to be drawn between a womb which is altogether small, being imperfectly developed, and a womb which is well developed excepting in having a contracted orifice. The former cases are allied to amenorrhœa; the latter are essentially cases of impeded menstruation.

4. That in this latter class of cases, which in practice will be found to be *very rare*, a perfect cure may be obtained by mechanical dilatation of the cervix.

5. That division of the cervix and the passage of a metallic dilator, afford an easy expedient for this purpose.

The second form of dysmenorrhœa is more common than that just mentioned, and has been well described by many writers on female diseases; but Dr. Oldham thinks a very material point in its history has generally been omitted, viz: the effect of its continuance in permanently enlarging and displacing the womb. The following case, which we shall abridge, exemplifies this variety of dysmenorrhœa:

A female, æt. 31 years, last living child aged 9 years, has since aborted several times at the fifth or sixth week; last miscarriage dates a twelve month back; since this has suffered from pain in the hips and back. When seen by Dr. Oldham, had a distressed countenance; complained of habitual pain at the sacrum, running down to the anus; pain in the right inguinal canal; occasional dysuria, and other pelvic symptoms, much increased by walking. Pain on sitting down, copious thick discharge; pain in coition, so severe as to cause its being given up. Menstruation regular but excessively painful, accompanied by clots and shreds of membrane. On examination the vagina was found hot and bathed with discharge; the uterus had prolapsed to within two inches of the external parts; the os was patent and uneven, and somewhat tilted under the pubes, the fundus being at the same time correspondingly retroverted. By speculum the cervix showed a surface of vivid granulations.

The treatment consisted in scarifying the cervix, taking about two ounces of blood at a time; perfect rest, and the use of saline aperients, and the following injection: decoct. papav. ℥vj; ext. conii, ℈j; liq. plumbi, ʒij. This plan was followed by considerable relief.

Subsequently to this period, the patient exhibited several specimens of dysmenorrhœal membrane, the passage of which was, as before, accompanied by severe pain. Being again placed under treatment, Dr. Oldham leeched the uterus once a week, and put her under a course of mercury, from which she has received considerable benefit. Dr. O. goes on to observe.

This is a case which, in its general features, is very frequently met with in practice, and may be very well taken to illustrate the pathology, symptoms, and treatment of membranous dysmenorrhœa. It will be seen that painful menstrual periods, with copious, rather than scanty flow, the casting off of portions of membrane, and then an increase in size and weight of the womb leading to its displacement, are the principal signs to be noticed. Partial prolapse, congestion, ulceration, and sterility may also be remarked.

The membrane which is thrown off in this disease varies in appearance, being sometimes a clear tubular cast of the uterine cavity. It more frequently, however, consists of shreds the size of the finger nail, or larger irregular pieces, the expulsion of which causes great suffering. Sometimes the menstrual blood coagulates round a portion of the membrane, and comes away as a hard compact clot. Very often the discharge consists of thin thread-like portions; at others, of half a cupful of thick tufts resembling the villi of the chorion filled with blood. The latter variety of discharge has, I believe, been often mistaken for the so called hydatid degeneration of the placenta; and I feel persuaded never occurs but when impregnation has preceded.

Dr. Oldham's views as to the formation of the dysmenorrhœal membrane are thus expressed.

It is generally thought to be lymph, but if some good specimens are carefully examined, they will be found to possess the same structure as the decidua. Not only do they resemble this structure in having a rough, attached, and a smooth free surface, but, what is more significant to their identity is, they are full of little holes and epithelium scales, which I do not doubt are the openings, and epithelium of the follicles of the uterine glands. It is very true, as Dr. Montgomery has noticed, that the small cotyledonous sacs are wanting, but this is often the case in true decidua thrown off in abortion. I doubt whether the swollen uterine glands bulge into sacs until the choreal villi are attached within them.

The practical bearing of the above remarks is, that the membranous exudation of dysmenorrhœa, equally with the decidua, depends upon an action commencing in the ovary. In healthy menstrution, Dr. Oldham observes that the various stages, the congestion of the ovary, the engorgement of the womb, and the flux are in harmony; but if the sexal appetite is excited in whatever way, then the uterine glands enlarge by sympathy, and a new formation begins, which is cast off at the next monthly period. As a sequel to the membranous forms of dysmenorrhœa, Dr. Oldham mentions retroversion of the womb.

I can bear testimony, he observes, to the truth of an observation of Dr. Rigby, that retroversion is one of the most common affections of the unimpregnated womb, and I would add that one of its common causes is the continuance of this membranous dysmenorrhœa. * * * The change occurs slowly, and takes several months to accomplish. The texture of the womb becomes altered. In a recent congestion the posterior wall is soft, compressible, and painful to the touch, but after repeated engorgements becomes harder, more solid, and very much like a fibrous growth.

I lay stress upon the swelling of the posterior wall, because it is more sensibly affected by congestion than the anterior wall. The natural convexity of this part becomes still more prominent, and when examined by the finger it often feels so round and solid, and swells out so abruptly from the cervix, that I am quite sure it is often mistaken for fibrous tumor. This swelling of the posterior wall forms a good practical distinction between a womb enlarged by congestion and one distended by early pregnancy. I have been in the habit of depending very much on the even enlargement of the anterior wall as a good diagnostic of early pregnancy.

The principal symptoms of the large and retroverted womb (incidental to this disease) are an additional weight in the lower part of the abdomen, and a painful sense of pressure about the sacrum; pain referred distinctly to the inguinal canals. There is pain also on sitting down, with a feeling as if some body were pressed upwards.

In the treatment of this condition of things, Dr. Oldham deprecates attempts to redress the displaced womb by the metallic sound. He relies upon keeping the patient quiet, regulating the diet, and small doses of mercury so as slightly to affect the gums. Leeches are applied to the cervix once or twice a week. Scarification is stated to be only useful when the cervix is granular and bleeds readily. Blisters to the sacrum are also useful. The liquor potassæ arsenitis, given as directed by Mr. Hunt, on a full stomach, is a good auxiliary after mercurial action has been obtained. By this means many supposed cases of fibrous degeneration of the uterus have been cured.

The conclusions to this paper are the following:

1. There is a form of menstruation rendered extremely painful from the production and casting off of a membrane from the cavity of the womb.

2. That this membrane is not the product of inflammation, or a thick mass of epithelium, but it is formed from the uterine glands, just as the decidua is, and is expelled in the same way.

3. That the morbid action does not begin at the uterus, but in the ovary, and the sequence of effects is, first, ovarian congestion, calling forth a sympathetic growth of the uterine glands, forming a false decidua, and uterine engorgement.

4. That this uterine engorgement is oftentimes relieved by a profuse menstrual flux; but if not, the posterior wall of the womb gradually increases in size and becomes hard; the balance of the organ is lost, and it becomes retroverted.

5. That the swelling of the posterior wall and falling back of the womb form a differential diagnosis between congestion and early pregnancy, the anterior wall enlarging in the latter, and the body being directed forwards.

6. That the symptoms of retroverted womb from this cause are not often mechanical obstruction to the other vicera.

7. That the treatment consists in strict attention to general health, leeching, and mercury.—*Medical Gazette.—Ranking's Abstract.*

